# Mechanisms for Maintaining Eukaryotic Replisome Progression in the Presence of DNA Damage

**DOI:** 10.3389/fmolb.2021.712971

**Published:** 2021-07-06

**Authors:** Thomas A. Guilliam

**Affiliations:** Division of Protein and Nucleic Acid Chemistry, Medical Research Council Laboratory of Molecular Biology, Cambridge, United Kingdom

**Keywords:** DNA replication, replisome, DNA damage tolerance, translesion synthesis, repriming, recoupling, replication fork

## Abstract

The eukaryotic replisome coordinates template unwinding and nascent-strand synthesis to drive DNA replication fork progression and complete efficient genome duplication. During its advancement along the parental template, each replisome may encounter an array of obstacles including damaged and structured DNA that impede its progression and threaten genome stability. A number of mechanisms exist to permit replisomes to overcome such obstacles, maintain their progression, and prevent fork collapse. A combination of recent advances in structural, biochemical, and single-molecule approaches have illuminated the architecture of the replisome during unperturbed replication, rationalised the impact of impediments to fork progression, and enhanced our understanding of DNA damage tolerance mechanisms and their regulation. This review focusses on these studies to provide an updated overview of the mechanisms that support replisomes to maintain their progression on an imperfect template.

## Introduction

In eukaryotes, DNA replication is initiated from multiple origins across the genome (Robinson and Bell, 2005). Here, MCM double hexamers—loaded onto duplex DNA in G1 phase of the cell cycle—are activated upon entry into S phase in a highly regulated manner, facilitated by the recruitment of numerous “firing factors” (Douglas et al., 2018). Many other origins remain dormant and only fire in response to replication impediments (Courtot et al., 2018). During activation, the lagging strand is ejected from each MCM ring to initiate unwinding of the parental duplex (Langston et al., 2017; Douglas et al., 2018), culminating in the formation of two active CMG (Cdc45-MCM-GINS) holo-helicases encircling their respective leading-strand templates (Lewis and Costa, 2020). Each CMG helicase translocates 3′–5′ on the leading strand to catalyse bidirectional fork propagation and additionally acts as a hub to coordinate the replicative enzymes and binding partners that collectively compose the replisome (Gambus et al., 2006; Fu et al., 2011; Pellegrini and Costa, 2016; Georgescu et al., 2017). Unwinding of the parental duplex provides a template for synthesis of nascent strands by the replicative polymerases (Pols) *α*, *δ*, and *ε* (Guilliam and Yeeles, 2020a). Since the DNA double helix is antiparallel and all DNA polymerases catalyse synthesis in the 5′–3′ direction, the nascent leading strand is synthesised mostly continuously in the same direction as replisome progression. Conversely, the lagging strand is replicated discontinuously in the opposite direction through repeated priming and extension to form Okazaki fragments. These are subsequently ligated together to generate a continuous daughter-strand molecule (Kainuma-Kuroda and Okazaki, 1975; Burgers and Kunkel, 2017).

To efficiently complete DNA replication, yeast replisomes must traverse inter-origin distances up to 100 kilobases (kb) before fork convergence and termination (Sekedat et al., 2010; Dewar and Walter, 2017). To cover such distances within S phase, replisomes progress at an average rate of 1.5–2 kb min^−1^ (Conti et al., 2007; Sekedat et al., 2010; Yeeles et al., 2017). However, genotoxic stress including DNA damage, DNA secondary structures and repeat sequences, interstrand and DNA-protein crosslinks, and limiting nucleotide levels can slow or stall replisome progression by inhibiting template unwinding and/or DNA synthesis (Zeman and Cimprich, 2014). Since CMG encircles and tracks along the leading strand, bulky obstacles located only in the lagging-strand template are more readily bypassed by the replisome (Fu et al., 2011). Similarly, during lagging-strand replication, polymerase stalling at one Okazaki fragment does not affect synthesis of subsequent fragments or replisome progression, resulting only in the generation of a single-stranded (ss) DNA gap (Taylor and Yeeles, 2018). Here, DNA damage tolerance (DDT) occurs in a postreplicative gap-filling manner to complete synthesis of the daughter strand molecule. In contrast, bulky leading-strand obstacles can block CMG translocation while cessation of leading-strand synthesis significantly slows the replisome (Fu et al., 2011; Taylor and Yeeles, 2018, 2019). This necessitates the coordination of DDT mechanisms with the replisome to maintain canonical replication fork progression in the presence of leading-strand template aberrations.

In recent years, structural, biochemical, and single-molecule approaches have begun to decipher the architecture and mechanism of unperturbed replisome progression, the effect of impediments on this, and the details of DDT mechanisms used to maintain progression. This review highlights these recent studies to provide an overview of DDT mechanisms which maintain or restart efficient CMG progression following slowing or stalling of the replisome.

## Replisome Architecture and Unperturbed Progression

**FIGURE 1:**
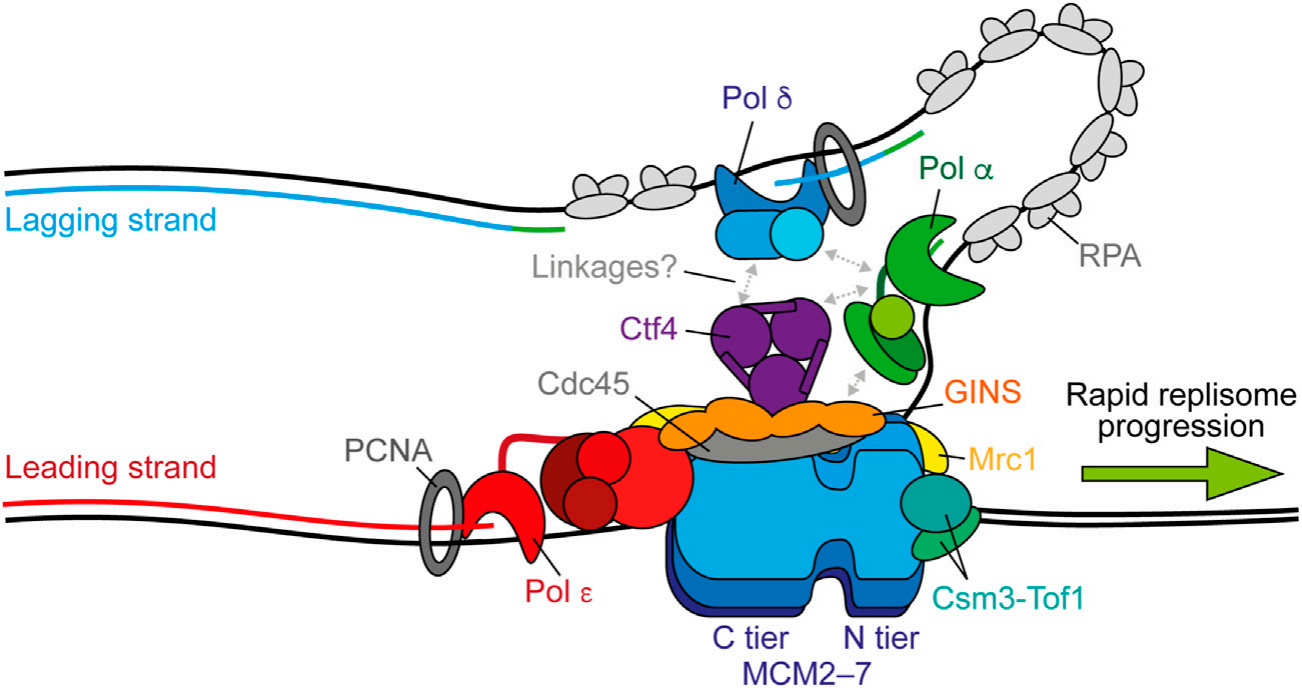
Replisome architecture during unperturbed progression. CMG (Cdc45-GINS-MCM2-7) tracks along the leading strand to unwind the parental duplex. Pol *ε* synthesises the leading strand continuously in complex with CMG. Pol *α* initiates discontinuous lagging-strand synthesis through regular priming. Primers are extended by Pol *δ* to form Okazaki fragments that are ligated together to produce a continuous daughter strand. Possible physical links between Pol *α*, Pol *δ*, Ctf4 (And-1), and CMG are indicated by gray arrows. Both Pol *ε* and Pol *δ* associate with PCNA during synthesis and RPA binds any exposed ssDNA. Csm3-Tof1(Tipin-Timeless) bind ahead of CMG and contact the parental duplex. Mrc1 (Claspin) may bind across one side of CMG spanning the N and C tiers. Synthesis by Pol *ε*, Pol *δ*, and Pol *α* is shown in red, blue, and green respectively.

The driving force of replisome progression is the ATP-dependent DNA unwinding activity of MCM, a two-tiered hetero-hexameric ring of Mcm2–7 subunits. One tier is composed of amino-terminal domains (N tier) and the other of carboxy-terminal AAA + ATPase domains (C tier), with ATP-binding sites located at the subunit interfaces, which serve as the motor to power DNA template unwinding (Bell and Botchan, 2013). Loading of Cdc45 and GINS—a hetero-tetramer of Psf1–3 and Sld5—on to MCM during initiation forms the CMG holo-helicase and stabilises the N tier (Figure 1) (Costa et al., 2011; Yuan et al., 2016). Following the reconstitution of origin-dependent eukaryotic DNA replication with purified yeast proteins (Yeeles et al., 2015), 2D single-particle electron microscopy (EM) studies of reconstituted CMG revealed that the helicase translocates with an N tier first orientation (Douglas et al., 2018). This is consistent with the orientation suggested by a previous 3D cryo-EM structure (Georgescu et al., 2017) and that observed in more recent studies (Goswami et al., 2018; Eickhoff et al., 2019; Yuan et al., 2020a; Baretić et al., 2020).

Helicase translocation results from the allosteric coupling of ATP hydrolysis to movement of DNA-binding elements in each subunit lining the centre of the ring (Enemark and Joshua-Tor, 2006). Structures of homo-hexameric helicases support a rotary translocation mechanism (Enemark and Joshua-Tor, 2006; Thomsen and Berger, 2009; Itsathitphaisarn et al., 2012; Gao et al., 2019; Meagher et al., 2019). Here, five-subunits form a right-handed staircase surrounding the DNA with the sixth disengaged. Sequential ATP binding, hydrolysis, and product release facilitate subunit movement to escort DNA between each subunit and through the staircase (reviewed in Enemark and Joshua-Tor, 2008). However, studies of CMG translocation are complicated by the observation that not all ATPase domains are equally important among the MCM subunits (Ilves et al., 2010; Eickhoff et al., 2019). Insight into this process has been offered by high resolution cryo-EM structures of *Drosophila* CMG in three translocation states during unwinding (Eickhoff et al., 2019). This study proposed an asymmetric hand-over-hand rotational mechanism of CMG translocation in which binding of Mcm5-Mcm3 to the ATPase staircase occurs as a block, promoting a double step. This could explain the functional asymmetry of ATPase domains in the MCM ring (Eickhoff et al., 2019). A recent cryo-EM structure of human CMG bound to fork DNA is consistent with a sequential rotary model of translocation, but the ATP-hydrolysis status of Mcm3 differs from the asymmetric translocation model proposed by Eickhoff et al. (2019). Further studies are necessary to resolve if these differences represent species-specific variation in the CMG translocation mechanism.

Regardless of the precise mechanistic details, the C tier motor ring pulls DNA through the N tier to facilitate strand separation and replisome translocation. In contrast to classic strand-exclusion models, where one strand is sterically excluded from the central channel of the helicase while the other is pulled through, duplex DNA enters the N tier of yeast CMG a short distance (Georgescu et al., 2017) and, moreover, the central channel is wide enough to completely accommodate duplex DNA (Wasserman et al., 2019). The N tier is subdivided into a zinc finger (ZF) ring at the front of CMG followed by an oligonucleotide-binding (OB) fold ring. A recent structure of yeast CMG revealed that separation of the parental duplex occurs at the bottom of the ZF sub-ring (Yuan et al., 2020a). Here, the lagging strand is diverted by the OB hairpin loops of Mcm3, Mcm4, Mcm6, and Mcm7, with a possible exit channel between the ZF domains of Mcm3 and Mcm5. This mechanism, referred to as the dam-and-diversion model, differs from the separation pin model, whereby a specific structural element separates the two strands. However, a putative strand-separation pin has been identified in a subsequent structure (Baretić et al., 2020). This is provided by a conserved phenylalanine in the N-terminal hairpin loop of Mcm7 that abuts the final base pair before strand separation (Baretić et al., 2020). Whether this conserved residue is required for efficient unwinding remains to be determined. In both structures, the path of the DNA template is the same and therefore separation may be achieved by a combination of the two mechanisms.

Although activated CMG helicases reconstituted from an origin *in vitro* are sufficient for robust DNA synthesis in the presence of Pol *α* and Pol *ε*, replication fork speeds are much slower than those observed *in vivo* in the absence of critical accessory proteins (Yeeles et al., 2015, 2017). These include the “fork protection complex” (FPC) comprised of Mrc1 (Claspin) and Csm3-Tof1 (Tipin-Timeless). A recent high resolution cryo-EM structure of the FPC and CMG on forked DNA revealed that Csm3-Tof1 bind to MCM via Tof1 at the front of the replisome (Figure 1) and grip the parental DNA duplex ahead (Baretić et al., 2020). Fork rate enhancement by Csm3-Tof1 is dependent on Mrc1 (Yeeles et al., 2017) which appears to bind across one side of CMG, spanning the N and C tiers (Baretić et al., 2020). The mechanism by which Mrc1 stimulates fork rates is not currently clear, however it also interacts with the flexible catalytic domain of Pol *ε* (Lou et al., 2008), potentially aiding its correct positioning behind CMG (Zhou et al., 2017). Pol *ε*—comprised of the catalytic subunit Pol2 and three non-catalytic subunits Dpb2, Dpb3, and Dpb4—is a core component of the replisome required for origin firing (Sengupta et al., 2013; On et al., 2014). It binds stably to CMG via interactions between the noncatalytic C-terminal domain of Pol2 with Mcm2 and Mcm5 (Goswami et al., 2018) and, additionally, between Dpb2—the accessory subunit of Pol *ε*—with Mcm3 and the GINS subunit Psf1 (Sengupta et al., 2013; Sun et al., 2015; Goswami et al., 2018; Yuan et al., 2020b). Dpb2 may also serve to direct the leading strand from CMG to the Pol *ε* active site (Yuan et al., 2020b). These interactions place Pol *ε* in complex with and behind CMG to couple leading-strand synthesis to template unwinding (Figure 1) (Guilliam and Yeeles, 2020a), which is essential for maximum fork rates (Yeeles et al., 2017; Taylor and Yeeles, 2018, 2019). The homo-trimeric eukaryotic sliding clamp processivity factor, PCNA, additionally helps tether the flexible catalytic domain of Pol2 to the nascent leading strand, further contributing to maximum replisome progression rates *in vitro* (Yeeles et al., 2017).

Unlike leading-strand synthesis, which is mostly continuous, lagging-strand replication requires repeated priming by Pol *α* and extension by Pol *δ* to generate Okazaki fragments (Figure 1) (Guilliam and Yeeles, 2020a). How Pol *α* is recruited to CMG to initiate primer synthesis is not currently understood. One potential mechanism is through Ctf4 (And-1), a trimeric hub that binds to CMG at the Cdc45-GINS interface (Yuan et al., 2019; Baretić et al., 2020; Rzechorzek et al., 2020) and interacts with a range of proteins including Pol *α* (Gambus et al., 2009; Simon et al., 2014; Villa et al., 2016; Guan et al., 2017; Kilkenny et al., 2017). However, Ctf4 is not required for DNA replication *in vitro* (Yeeles et al., 2015, 2017; Kurat et al., 2017) and only minimally affects retention of Pol *α* at the replisome in yeast cells (Kapadia et al., 2020). Instead, the Ctf4-Pol *α* interaction may be more important for recycling of parental histones on to the lagging strand, than for primer synthesis (Evrin et al., 2018; Gan et al., 2018). Curiously, a recent cryo-EM report demonstrated that Ctf4 can link two CMGs into a single “replication factory” (Yuan et al., 2019). However, further studies are required to confirm whether this occurs during active replication.

Until recently, Pol *δ* was assumed to function disconnected from the replisome, synthesising each Okazaki fragment while bound to PCNA before dissociating from DNA to permit ligation (Bell and Labib, 2016; Lancey et al., 2020a). However, two single-molecule studies, one *in vitro* (Lewis et al., 2020) and one *in vivo* (Kapadia et al., 2020), have challenged this assumption by demonstrating that Pol *δ* is retained at the replisome for multiple rounds of Okazaki fragment synthesis. It was suggested that Pol *δ* may interact with the replisome via Pol *α* (Huang et al., 1999; Johansson et al., 2004; Lewis et al., 2020). However, *in vivo* the residency time of Pol *δ* at the replisome exceeded that of Pol *α*, indicating that it interacts with CMG independently, potentially through Ctf4 (Figure 1) (Bermudez et al., 2010; Kapadia et al., 2020). Since lagging-strand synthesis occurs in the opposite direction to CMG progression, these findings additionally posit the existence of lagging-strand loops (Figure 1). Unlike Pol *ε*, Pol *δ* is not required for maximum rates of replisome progression, but can support leading-strand synthesis, albeit more slowly, in the absence of active Pol *ε* and is essential for complete lagging-strand synthesis (Yeeles et al., 2017). Consequently, although emerging evidence supports Pol *δ* as a stable component of the replisome, coupling of Pol *δ* to CMG does not appear to be important for maximum fork rates, likely because lagging-strand synthesis occurs in the opposite direction to replisome translocation. However, recent biochemical and cellular studies revealed that Pol *δ* also plays a key role in the initiation of leading-strand synthesis. Here, it extends the first lagging-strand primer, generated by the oppositely translocating replisome, back across the origin to couple synthesis to CMG-Pol *ε* and establish rapid fork progression (Garbacz et al., 2018; Aria and Yeeles, 2019).

## Uncoupling of Leading-Strand Synthesis and Fork Slowing

Since the central channel of CMG is wide enough to accommodate duplex DNA, many DNA lesions can readily pass through the helicase, stalling DNA synthesis instead of CMG translocation (Zeman and Cimprich, 2014). Damaged nucleobases, abasic sites, and more bulky adducts such as pyrimidine dimers can be accommodated in the central channel of CMG and therefore do not pose a steric block to unwinding even when present in the leading-strand template (Taylor and Yeeles, 2018, 2019; Guilliam and Yeeles, 2021). However, the replicative polymerases are typically considered to be intolerant to DNA lesions and mismatched 3′ termini (Sale et al., 2012). The general structure of replicative polymerases is likened to a right hand; the catalytic site sits in the palm domain while the finger and thumb domains grip the template and primer (Sale et al., 2012). The intolerance of replicative polymerases is in part due to “induced fit” conformational changes during catalysis, whereby the finger domain moves to switch the polymerase from an open to a closed conformation only when the incoming dNTP correctly pairs with the templating base (Johnson, 2008; Freudenthal et al., 2013; Yang and Gao, 2018). The recent structure of the Pol *ε* holoenzyme revealed that the finger domain tilts 27° upon DNA-dNTP binding, changing the enzyme from a gapped circle conformation to one that completely encircles DNA ready for catalysis (Yuan et al., 2020b). The compact catalytic site prevents the accommodation of bulky DNA adducts, while 3′ mismatches weaken DNA binding to the active site triggering relocation to the 3′–5′ proofreading exonuclease domain (Reha-Krantz, 2010). These features greatly improve fidelity and processivity but also make the enzyme intolerant to lesions which pass through CMG (Yang and Gao, 2018). Similarly, limiting nucleotide levels and repetitive DNA sequences can stall synthesis without directly stopping CMG translocation (Zeman and Cimprich, 2014; Devbhandari and Remus, 2020).

**FIGURE 2:**
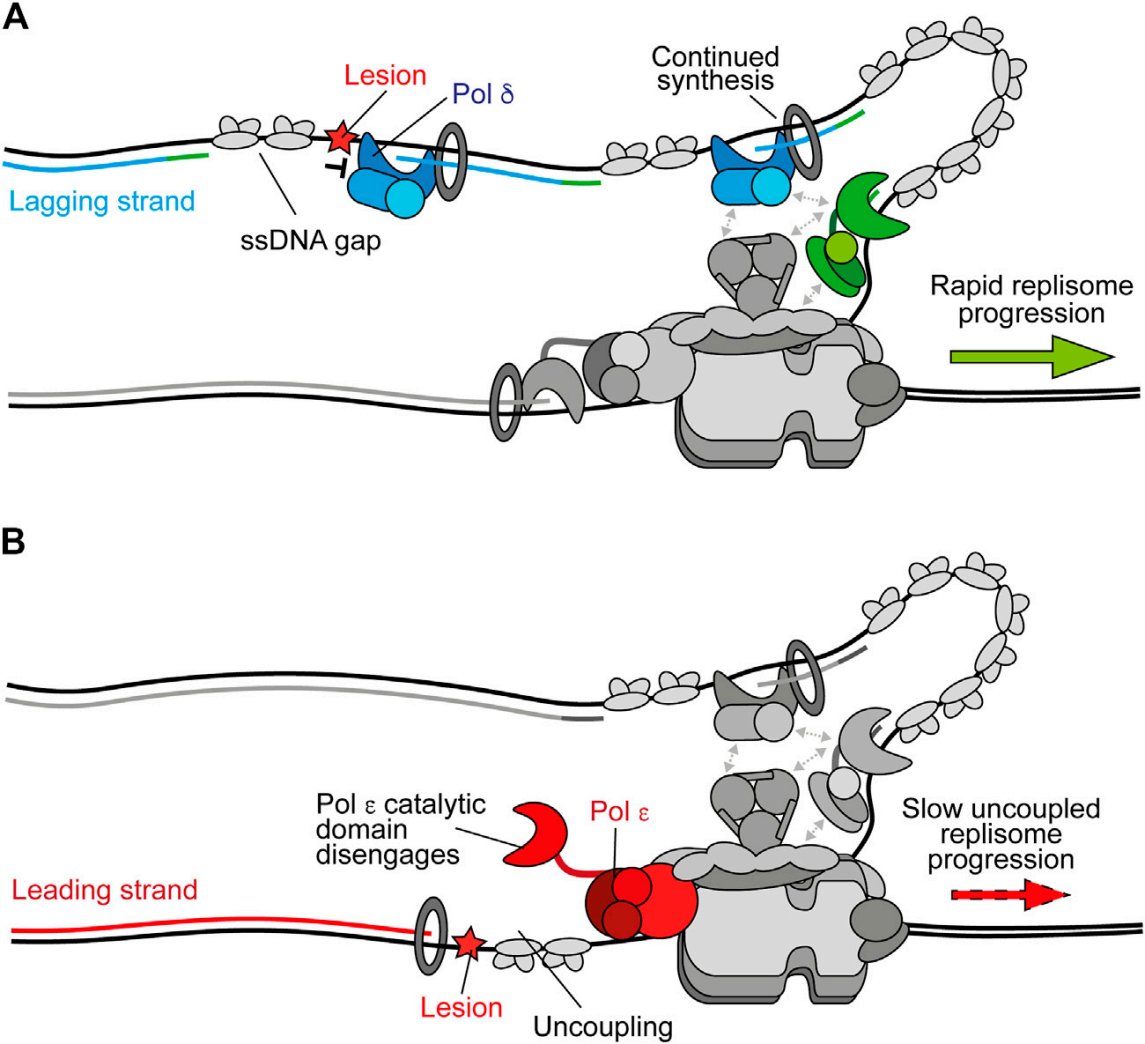
Polymerase stalling on the leading and lagging strands. **(A)** Stalling of Pol *δ* at a DNA lesion during synthesis of one Okazaki fragment does not affect synthesis of subsequent fragments or ongoing replication fork speeds. Instead, a persistent ssDNA gap is formed. Synthesis by Pol *δ* and Pol *α* are shown in blue and green respectively. Other replisome components are shown in gray for clarity **(B)** Stalling of Pol *ε* by a DNA lesion during leading-strand synthesis causes uncoupling due to disengagement of the flexible catalytic domain from the nascent strand and PCNA. Here, template unwinding and lagging-strand synthesis continue but at a much-reduced rate, resulting in the generation of RPA-coated ssDNA on the leading strand. Synthesis by Pol *ε* is shown in red.

Stalling of Pol *δ* on the lagging strand does not affect ongoing replisome progression or the synthesis of subsequent Okazaki fragments because priming occurs continually downstream, with respect to helicase translocation direction (Figure 2A) (Taylor and Yeeles, 2018). This leaves a short ssDNA gap which can be filled in behind the fork by classic DDT mechanisms (Leung et al., 2019). Moreover, Pol *δ* is not required for maximum fork rates (Yeeles et al., 2017). In contrast, stalling of Pol *ε* causes uncoupling of leading-strand synthesis from template unwinding, resulting in slow uncoupled fork progression (Figure 2B). Here, template unwinding and lagging-strand synthesis continue in the absence of leading-strand synthesis but at a much-reduced rate (Taylor and Yeeles, 2018, 2019). Uncoupled forks can progress multiple kilobases (Taylor and Yeeles, 2018, 2019), generating long stretches of RPA-coated ssDNA on the leading strand, as observed in UV-irradiated yeast cells (Lopes et al., 2006). It is likely that uncoupling occurs as a result of the dissociation of the flexibly tethered catalytic domain of CMG-bound Pol *ε* from the 3′ end of the nascent leading strand and PCNA (Figure 2B). This has been proposed to also occur spontaneously in the absence of genotoxic stress to populate the leading strand with PCNA for nucleosome assembly and mismatch repair (Georgescu et al., 2017; Zhou et al., 2017).

If not rapidly resolved, the RPA-coated ssDNA accumulated from uncoupling can activate the DNA replication checkpoint by providing a platform for recruitment of Mec1-Ddc2 in yeast, or ATR-ATRIP in vertebrates (Byun et al., 2005; Pardo et al., 2017). This subsequently activates the checkpoint effector kinase Rad53 in budding yeast, or CHK1 in vertebrates, to elicit the cellular stress response. In reconstituted DNA replication experiments, Rad53 was recently shown to further slow uncoupled CMG progression (Devbhandari and Remus, 2020). This may help prevent RPA depletion when uncoupling occurs for an extended period of time (Toledo et al., 2013). This mechanism was shown to be important in response to hydroxyurea (HU) which inhibits ribonucleotide reductase (RNR), resulting in dNTP depletion (Gan et al., 2017). In *rad53-1* mutant yeast cells, HU treatment caused asymmetric DNA synthesis whereby extension proceeded much further along the lagging strand than the leading strand as a result of uncoupling. This may be due to a requirement for higher dNTP levels for leading-strand synthesis by Pol *ε* than lagging-strand synthesis by Pol *δ*. Uncoupling in *rad53-1* cells was suppressed by elevated dNTP levels (Gan et al., 2017). Importantly, checkpoint kinases upregulate dNTP levels in response to replication stress (Yeeles et al., 2013). Rad53 may therefore be important for limiting uncoupling by both directly preventing excessive template unwinding by CMG and promoting recoupling by elevating dNTP levels. Recent biochemical data demonstrate that direct slowing of replication forks by Rad53 is at least in part mediated by Mrc1 (McClure and Diffley, 2021, Preprint). Although Mrc1 functions upstream of Rad53 following replication stress, it is also a target for phosphorylation by Rad53 and Mec1 (Alcasabas et al., 2001). Phosphorylation of Mrc1 prevents it from stimulating CMG, thereby slowing template unwinding (McClure and Diffley, 2021, Preprint).

Interestingly, inhibition of RNR by HU also elevates reactive oxygen species (ROS) in human cells (Somyajit et al., 2017). This can cause replication fork slowing via a checkpoint-independent mechanism. Here, peroxiredoxin 2 (PRDX2) acts as a ROS sensor. At low ROS levels it binds to Timeless in an oligomeric state. However, elevated ROS levels disrupt oligomerised PRDX2, causing its dissociation and the displacement of Timeless from the replisome, consequently slowing fork progression (Somyajit et al., 2017).

## Replicase Switching and Recoupling

**FIGURE 3:**
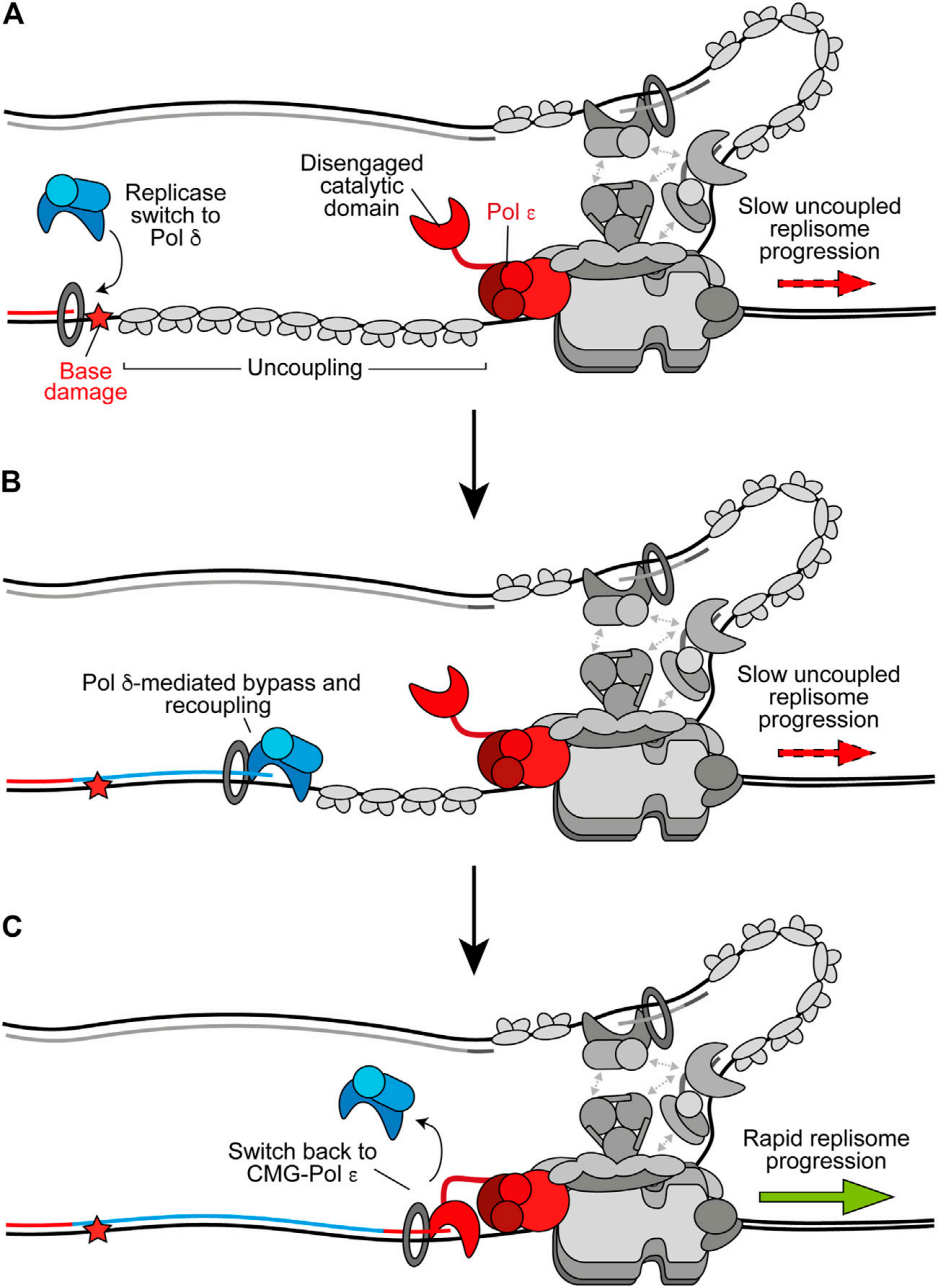
Replicase switching and recoupling of leading-strand synthesis. **(A)** Upon uncoupling, a replicase switch occurs. Here, Pol *δ* is recruited to the uncoupled nascent leading strand. Other replisome components are shown in gray for clarity. **(B)** When stalling of Pol *ε* occurs due to any factor that is more readily tolerated by Pol *δ*, such as certain oxidative lesions, Pol *δ* directly extends the nascent leading strand through the impediment to recouple synthesis. **(C)** Upon recoupling the nascent leading strand is handed back to CMG-bound Pol *ε* to restore rapid replisome progression. Synthesis by Pol *ε* and Pol *δ* is shown in red and blue respectively.

Accumulating evidence suggests that, in addition to its role as the lagging-strand replicase, Pol *δ* replicates the leading strand whenever synthesis is not coupled to CMG (Guilliam and Yeeles, 2020a). Consistent with this, Pol *δ* acts as a “first responder” to stalling of leading-strand synthesis by outcompeting other polymerases, including free Pol *ε*, for the uncoupled nascent strand (Figure 3A) (Guilliam and Yeeles, 2020b). Once recruited, Pol *δ* can readily recouple the leading strand to CMG-Pol *ε* whenever uncoupling occurs spontaneously or due to factors which are more readily tolerated by Pol *δ* than Pol *ε* (Figure 3B) (Guilliam and Yeeles, 2020b). Indeed, we recently showed that Pol *δ* can efficiently bypass the oxidative single base lesions, thymine glycol and 8-oxoguanine, to recouple uncoupled forks *in vitro* (Guilliam and Yeeles, 2021). Similarly, Pol *δ* promotes recoupling following stalling of leading-strand synthesis at hairpin-forming sequences (Casas-Delucchi et al., 2021, Preprint). In conjunction, studies of vertebrate cells support a role for Pol *δ* in bypassing some lesions at the replication fork (Hirota et al., 2015, 2016). This replicase switch mechanism allows the replisome to quickly resume rapid progression following uncoupling, limiting ssDNA exposure on the leading strand while maintaining synthesis by the high-fidelity replicative polymerases; in turn avoiding potentially mutagenic DDT mechanisms or checkpoint activation. Similarly, Pol *δ* can excise and correct errors made by Pol *ε*, whereas Pol *ε* can only correct its own misincorporations (Flood et al., 2015; Bulock et al., 2020). This suggests that misincorporated nucleotides that are not removed by Pol *ε* may cause uncoupling, facilitating a switch to Pol *δ* before proofreading of the error and recoupling of leading-strand synthesis. A recent cryo-EM structure of the yeast Pol *δ* holoenzyme revealed a high degree of flexibility between the Pol3 catalytic and Pol31–Pol32 regulatory subunits, which may aid proofreading and facilitate binding to a wider variety of DNA substrates (Jain et al., 2019).

Pol *δ* is likely favored for recoupling due to its faster rate of synthesis than the rate of uncoupled CMG progression, higher affinity than free Pol *ε* for PCNA, and its ability to processively synthesise kilobases of DNA on an RPA-coated template in the presence of PCNA (Langston and O’Donnell, 2008; Georgescu et al., 2014; Yeeles et al., 2017; Sparks et al., 2019). Interestingly, a structure of Pol *δ*-PCNA recently demonstrated that the polymerase holds the DNA substrate such that it is positioned through the centre of the PCNA clamp without direct PCNA-DNA contacts (Zheng et al., 2020). DNA-clamp interactions are instead mediated by water, allowing PCNA to function as a “water skate” to permit rapid and processive DNA synthesis while generating minimal resistance. Likewise, PCNA was recently shown to enhance nucleotide incorporation rates by Pol *δ* from 40 nucleotides per second to more than 350 per second (Mondol et al., 2019). This rate of nucleotide incorporation would allow the polymerase to rapidly catch up with uncoupled CMG, which has been estimated to unwind DNA at ∼55 base pairs per minute (Sparks et al., 2019; Devbhandari and Remus, 2020). It is not currently understood how the nascent leading strand is transferred from Pol *δ* back to CMG-Pol *ε* (Figure 3C). However, unlike free Pol *ε*, CMG-Pol *ε* does outcompete Pol *δ* for the leading strand (Georgescu et al., 2014). It is possible handover occurs through collision release in which Pol *δ* is ejected from PCNA upon running into uncoupled CMG, similar to the mechanism proposed to occur during Okazaki fragment synthesis (Langston and O’Donnell, 2008; Schauer and O’Donnell, 2017). Regardless, rapid fork rates are reinstated following Pol *δ*-mediated recoupling, suggesting CMG-Pol *ε* efficiently resumes leading-strand synthesis downstream (Guilliam and Yeeles, 2020b).

## Translesion Synthesis Polymerases

**FIGURE 4:**
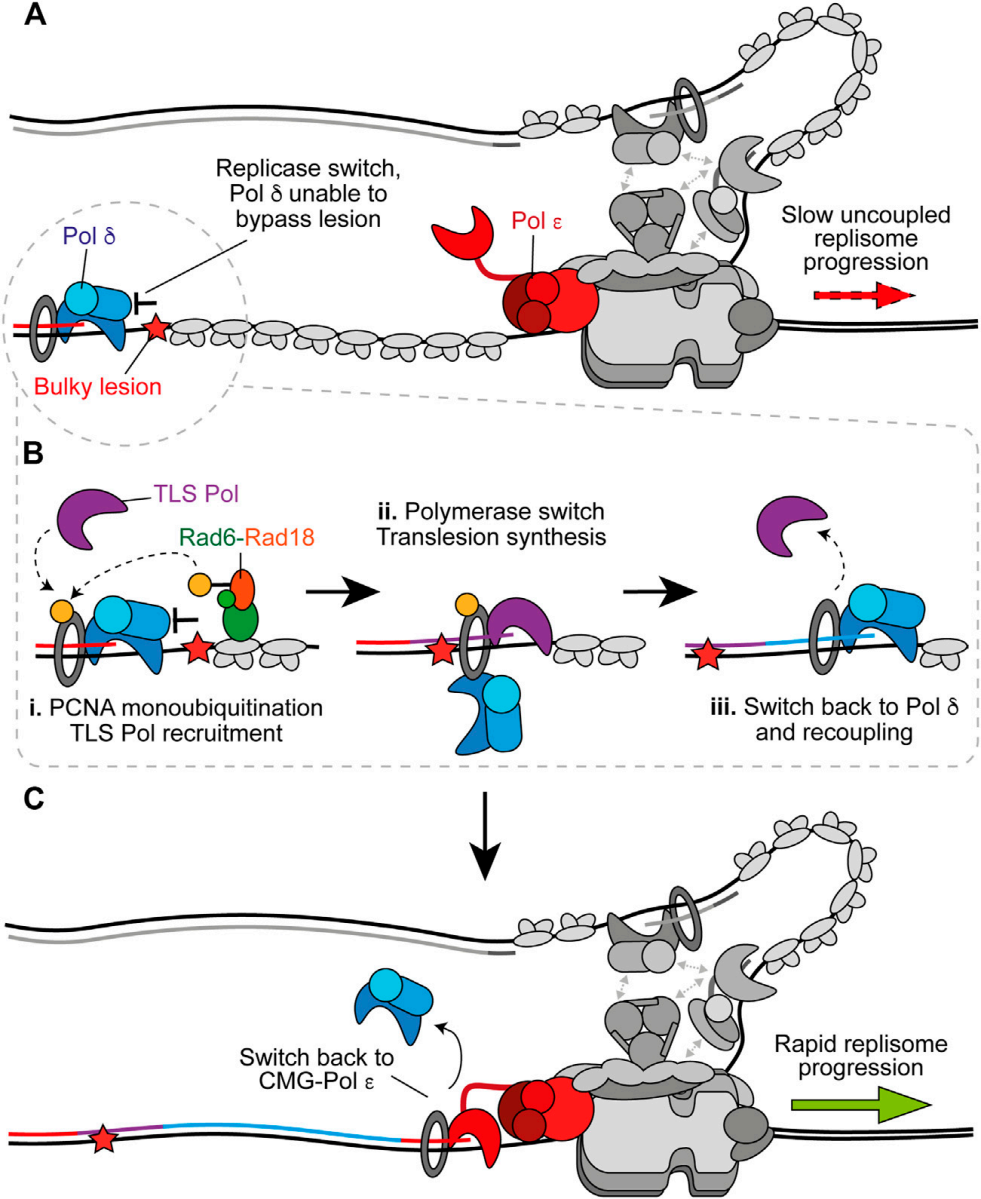
On the fly translesion synthesis can promote recoupling. **(A)** When Pol *δ* is recruited to the leading strand following uncoupling, but is unable to bypass the impediment, TLS is required prior to recoupling. Other replisome components are shown in gray for clarity. **(B)** i. RPA-coated ssDNA recruits Rad6-Rad18 which catalyse monoubiquitination of PCNA. This stimulates recruitment of a TLS polymerase. ii. The TLS polymerase extends the nascent strand past the impediment. This could occur without complete dissociation of Pol *δ* from PCNA. iii. After bypass a switch back to Pol *δ* facilitates rapid recoupling. **(C)** Pol *δ* hands the nascent leading strand back to Pol *ε* to restore canonical fork rates. Synthesis by Pol *ε*, Pol *δ*, and the TLS polymerase is shown in red, blue, and purple respectively.

Despite a greater ability to tolerate certain single-base lesions than Pol *ε* (Guilliam and Yeeles, 2021), Pol *δ* also stalls upon encountering more bulky or distorting lesions such as a cyclobutane pyrimidine dimer (CPD), a well-characterised UV-induced photoproduct (Taylor and Yeeles, 2018; Guilliam and Yeeles, 2020b). In these instances, specialised translesion synthesis (TLS) polymerases are required for direct synthesis across damage prior to recoupling (Guilliam and Yeeles, 2020b). TLS that functions to promote recoupling is termed “on the fly” TLS (Figure 4) to distinguish it from gap-filling TLS. The main TLS polymerases in eukaryotes are those of the Y-family; Pol *η*, Pol *ι*, Pol *κ*, and Rev1, in addition to the B-family polymerase Pol *ζ* (Sale et al., 2012). Of these, only Pol *η*, Rev1, and Pol *ζ* are present in budding yeast. The Y-family polymerases have spacious solvent accessible active sites formed between the palm and finger domains that make few, mostly non-specific, contacts with the DNA template, permitting the accommodation of bulky lesions and template distortions (Fleck and Schär, 2004; Sale et al., 2012). They also possess an additional “little finger” domain, not present in other polymerase families, which together with the thumb domain grips duplex DNA and may play a role in lesion specificity (Boudsocq et al., 2004; Fleck and Schär, 2004; Wilson et al., 2013). Compared to the replicative polymerases, the finger domain of the Y-family polymerases is stubbier and remains in a closed conformation irrespective of DNA-dNTP binding, thereby removing the “induced fit” mechanism of dNTP selection (Vaisman and Woodgate, 2017). They also lack proofreading exonuclease activity and therefore the ability to excise mispaired nucleotides. Consequently, the Y-family polymerases gain an increased tolerance of template lesions at the expense of fidelity and processivity.

Pol *ζ* is thought to function as an extender when two polymerases are required for lesion bypass. Here, a Y-family polymerase first incorporates nucleotides opposite the damage, before Pol *ζ* takes over to catalyse extension beyond the lesion in a process that is typically error-prone (Johnson et al., 2000). Only recently was the structure of Pol *ζ* determined (Malik et al., 2020), revealing that it adopts a pentameric ring conformation composed of the catalytic subunit Rev3, two Rev7 subunits, and the non-catalytic Pol31 and Pol32 subunits which are shared with Pol *δ* (Johnson et al., 2012; Makarova et al., 2012). Similar to the replicative polymerases, the finger domain transitions from an open to a closed conformation upon binding the correct dNTP, conferring higher fidelity during nucleotide selection compared to the Y-family polymerases (Malik et al., 2020). However, key differences between Pol *ζ* and Pol *δ* explain the former’s penchant for extending aberrantly paired 3′ ends (Johnson et al., 2000). In Pol *δ*, the linker between the NTD and palm domain contacts the terminal base pair to prevent efficient extension of a mismatch. However, these contacts are not present in Pol *ζ* and although the polymerase contains an exonuclease domain, it is inactive. These features make Pol *ζ* well suited to fulfill an extender role during TLS and reveal why, despite having higher fidelity than the Y-family polymerases (McCulloch and Kunkel, 2008), it is involved in almost all damage-induced mutagenesis (Lawrence, 2002).

## The Polymerase Switch During TLS

On the fly TLS can rescue uncoupled forks when Pol *δ* alone is unable to recouple the leading strand (Figure 4A) (Guilliam and Yeeles, 2020b). However, Pol *δ* outcompetes TLS polymerases for binding to stalled nascent 3′ ends and in doing so limits mutagenesis by negatively regulating TLS when it is not required for recoupling (Guilliam and Yeeles, 2020b). RPA-coated ssDNA, generated by persistent stalling, recruits the E2–E3 ubiquitin ligase complex, Rad6–Rad18 (Watanabe et al., 2004; Davies et al., 2008), which facilitates monoubiquitination of PCNA to promote a switch from Pol *δ* to TLS polymerase to facilitate lesion bypass (Figure 4B) (Hoege et al., 2002; Stelter and Ulrich, 2003; Bienko et al., 2005; Garg and Burgers, 2005; Plosky et al., 2006; Guilliam and Yeeles, 2020b). Y-family TLS polymerases are recruited to monoubiquitinated PCNA via their ubiquitin-binding ZF (Pol *η* and Pol *κ*) or helical ubiquitin-binding motifs (Pol *ι* and Rev1), in addition to canonical PCNA interacting peptide (PIP) box motifs in their non-catalytic C-terminal extensions (Leung et al., 2019). Rev1 lacks a PIP box and instead interacts with PCNA via its N-terminal BRCT and little finger domains (Guo et al., 2006; Sharma et al., 2011).

The ubiquitin moiety is located on the back side of PCNA (Freudenthal et al., 2010). This may allow TLS polymerases to be recruited to the back face of monoubiquitinated PCNA while Pol *δ* remains bound at the front (Freudenthal et al., 2010). This toolbelt model, in which multiple binding partners can occupy PCNA monomers not bound by Pol *δ*, is supported by the recent structure of Pol *δ*-PCNA-FEN1 in complex on DNA (Lancey et al., 2020a) which suggests a toolbelt mechanism for flap cleavage during Okazaki fragment maturation. This study also revealed that PCNA can tilt up to 20°, disrupting interactions between the catalytic subunit of Pol *δ* and PCNA that are critical for DNA synthesis, while the polymerase remains bound to PCNA via the PIP box located in its thumb domain (Lancey et al., 2020a). The same group recently solved the structure of Pol *κ*-PCNA-DNA and stalled Pol *δ*-PCNA-DNA to propose a mechanism of polymerase switching (Lancey et al., 2020b, Preprint). Therein, Pol *δ* releases the primer-template from the active site upon stalling, Pol *κ* binds to an exposed PCNA protomer in a flexible state and either actively displaces Pol *δ*, or Pol *δ* independently dissociates, from DNA to form the final Pol *κ* holoenzyme. Importantly, tilting of PCNA provides enough room to accommodate active Pol *κ* and retain Pol *δ* on PCNA via its thumb domain PIP box. Pol *δ* might therefore hand over synthesis to the TLS polymerase without dissociating from PCNA to allow a rapid switch back after lesion bypass (Figure 4B), before subsequent recoupling (Figure 4C). Importantly, evidence supports a toolbelt model of polymerase switching in *E. coli* (Indiani et al., 2005; Kath et al., 2016) and archaea (Cranford et al., 2017), giving credence to the prospect that a similar mechanism occurs in eukaryotes. However, it is also possible that Pol *δ* fully dissociates from PCNA and DNA to permit TLS before reassociating to recouple synthesis.

In addition to PCNA, the C-terminal domain of Rev1 interacts with the other Y-family TLS polymerases through PIP-like motifs (Boehm and Washington, 2016), and binds the Rev7 subunit of Pol *ζ* (Murakumo et al., 2001; Zhao and Washington, 2017). Numerous studies support a non-catalytic role for Rev1 in the recruitment of other TLS polymerases (Nelson et al., 2000; Haracska et al., 2001; Ross et al., 2005; Edmunds et al., 2008). One proposed mechanism is through the formation of “Rev1 bridges” in which a TLS polymerase is linked to PCNA via Rev1 without interacting directly with the clamp. Both PCNA toolbelts and Rev1 bridges have been observed in single-molecule studies and they can interchange dynamically without dissociation (Boehm et al., 2016). The relative contribution of PCNA monoubiquitination and Rev1 in recruiting and coordinating TLS is likely to be lesion specific (Wang and Xiao, 2020). Indeed, accurate CPD bypass by Pol *η* does not require Rev1 or Pol *ζ* but is dependent on PCNA monoubiquitination (Kannouche et al., 2004; Bienko et al., 2005; Andersen et al., 2011; Guilliam and Yeeles, 2020b), whereas BaP-dG (*N* ^*2*^-benzo[a]pyrene-dG) bypass by Pol *κ* is dependent on its interaction with Rev1 (Ohashi et al., 2009). The requirement for Rev1 is more important for two-polymerase TLS that also requires Pol *ζ* for extension and is typically error-prone (Livneh et al., 2010). Moreover, the affinity of different TLS polymerases for Rev1 varies, in some cases being relatively weak and allowing competition (Pustovalova et al., 2016). Consequently, Rev1 may help select a different polymerase if the first cannot bypass the lesion following recruitment via monoubiquitinated PCNA, switching TLS from a one to a two-polymerase mode where necessary. In addition to Rev7, Rev1 was more recently found to also interact with the PolD3 (Pol32 in yeast) subunit of human Pol *ζ*/Pol *δ* with higher affinity than it does with other Y-family polymerases (Pustovalova et al., 2016). This interaction may help displace the “inserter” polymerase from Rev1 and monoubiquitinated PCNA to facilitate extension by Pol *ζ* beyond the lesion (Pustovalova et al., 2016). The sharing of Pol31-Pol32 between Pol *δ* and Pol *ζ* led to the proposal that removal of Pol3 and recruitment of Rev3-Rev7 at the site of damage coordinates a switch between the two (Baranovskiy et al., 2012). In support, Pol3 is degraded in response to damage (Daraba et al., 2014). However, Pol *ζ* exists as a stable four-subunit complex (Rev3–Rev7–Pol31–Pol32) in all stages of the cell cycle irrespective of damage, potentially arguing against subunit exchange with Pol *δ* (Makarova et al., 2012).

## Repriming

Aside from TLS, uncoupled replication forks can be rescued by reinitiating leading-strand synthesis through the generation of a new primer downstream of the impediment in a process termed repriming. Similar to ongoing Okazaki fragment synthesis after Pol *δ* stalling on the lagging strand, this leaves behind a ssDNA gap which can be filled by TLS or template switching. In reconstitution experiments with yeast proteins, Pol *α* could readily synthesise primers on the lagging strand but was inefficient at repriming the leading strand due to inhibition by RPA (Taylor and Yeeles, 2018). This disparity between leading and lagging-strand priming efficiency suggests these two processes are mechanistically distinct. It is likely that lagging-strand priming is facilitated by the position of Pol *α* in the replisome, while leading-strand repriming uses free Pol *α* and is therefore sensitive to RPA concentrations. Coupling of leading-strand synthesis during initiation occurs via extension of a lagging-strand primer back across the origin by Pol *δ* (Garbacz et al., 2018; Aria and Yeeles, 2019), further arguing against an efficient Pol *α*-mediated leading-strand priming mechanism in unperturbed conditions. However, it is possible that leading-strand repriming by Pol *α* occurs under conditions of RPA exhaustion, or requires factors or modifications not present in the reconstituted system (Lopes et al., 2006; Fumasoni et al., 2015; Toledo et al., 2017; Taylor and Yeeles, 2018).

**FIGURE 5:**
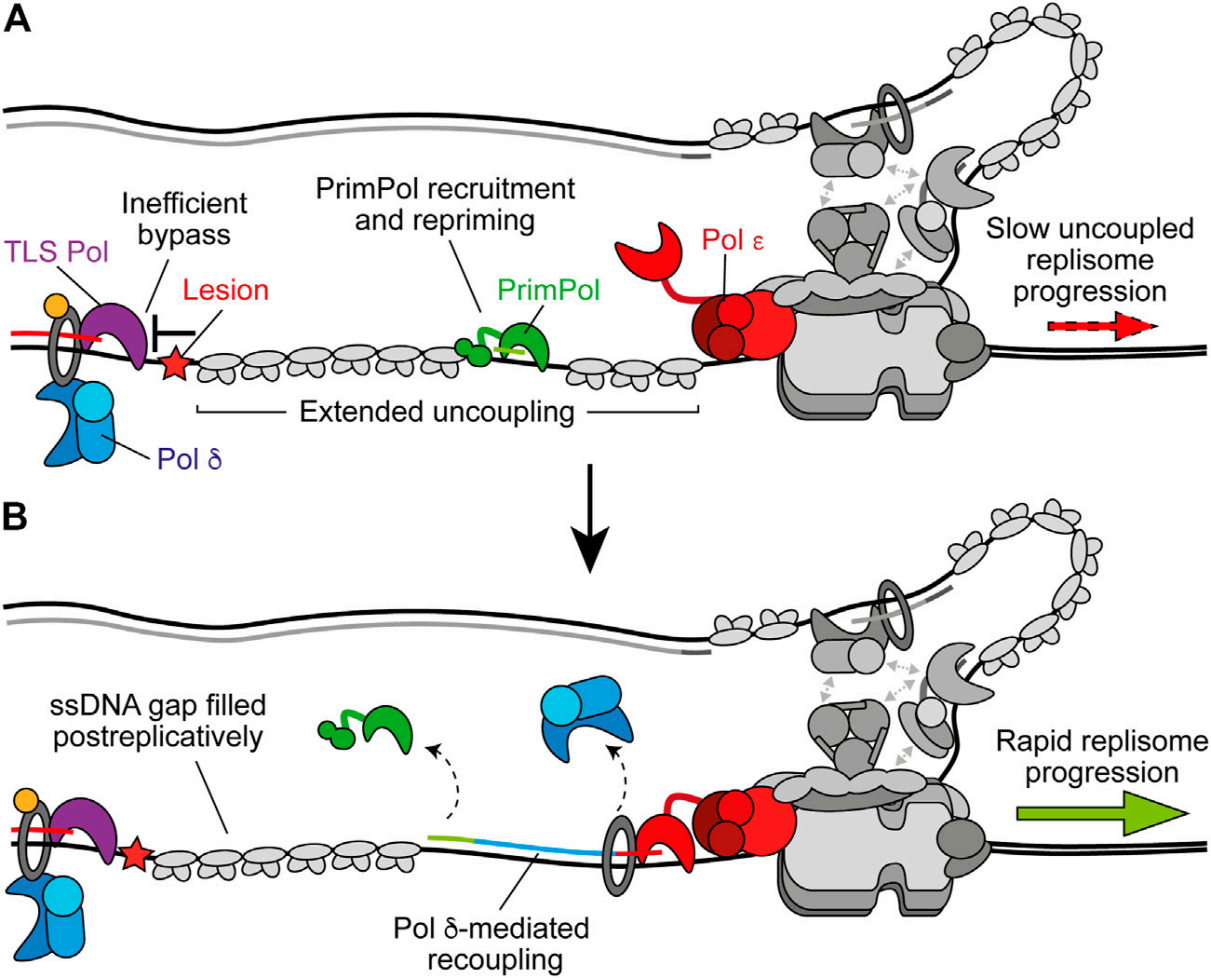
PrimPol-mediated repriming restarts leading-strand synthesis. **(A)** When replicase switching and on the fly TLS are inefficient at bypassing the leading-strand impediment, extended uncoupling occurs. This stimulates the recruitment of PrimPol to RPA-coated ssDNA downstream and subsequent repriming. Other replisome components are shown in gray for clarity. **(B)** Following repriming, Pol *δ* may extend the nascent primer to recouple synthesis to Pol *ε* and restore rapid replisome progression rates. This leaves behind a ssDNA gap at the site of the lesion which may be filled in postreplicatively by TLS or template switching. Synthesis by Pol *ε*, Pol *δ*, and PrimPol are shown in red, blue, and green respectively.

In higher eukaryotes, leading-strand repriming is catalysed by a second archaeo-eukaryotic primase named PrimPol that is absent in budding yeast (Bianchi et al., 2013; García-Gómez et al., 2013; Wan et al., 2013; Guilliam et al., 2015b; Guilliam and Doherty, 2017). PrimPol interacts directly with RPA and this interaction is required for recruitment of the primase to the ssDNA generated by uncoupling (Figure 5A) (Guilliam et al., 2015a, 2017; Šviković et al., 2019). The naming of PrimPol reflects its ability to perform both DNA primase and polymerase activities, and similar to the TLS polymerases it can directly bypass a number of lesions *in vitro* (Bianchi et al., 2013; Keen et al., 2014b). However, the catalytic domain of PrimPol does not resemble a canonical polymerase fold, completely lacks a thumb subdomain, makes almost no contacts with the primer strand, has very low inherent processivity, and does not noticeably interact with PCNA (Keen et al., 2014b; Guilliam et al., 2015a; Rechkoblit et al., 2016). Likewise, although PrimPol can bypass a (6–4) T–T photoproduct, its active site is not able to accommodate this lesion and it is likely bypassed by a looping out mechanism that generates deletions (Bianchi et al., 2013; Rechkoblit et al., 2016; Guilliam and Doherty, 2017). Moreover, the primase but not polymerase activity of PrimPol is dependent on its noncatalytic C-terminal ZF domain, which binds and selects the first 5′-nucleotide of the nascent primer strand (Keen et al., 2014b; Martínez-Jiménez et al., 2018). An intact ZF is essential for PrimPol’s function *in vivo* (Mourón et al., 2013; Keen et al., 2014a, 2014b; Kobayashi et al., 2016; Schiavone et al., 2016; Šviković et al., 2019). Consequently, the available evidence strongly suggests the primary function of PrimPol *in vivo* is as a primase not a TLS polymerase (Guilliam and Doherty, 2017).

In avian cells, loss of PrimPol causes damage sensitivity, replication fork slowing and growth arrest after damage (Bailey et al., 2016; Kobayashi et al., 2016). In human cells, deletion of PrimPol does not affect survival after damage but does result in delayed recovery, increased mutagenesis, and sister chromatid exchanges (Bailey et al., 2019). However, loss of PrimPol in human cells lacking Pol *η* or Pol *ζ* significantly increases sensitivity to damage (Kobayashi et al., 2016; Bailey et al., 2019). This suggests that PrimPol may function to rescue uncoupled forks when Pol *δ* alone or TLS cannot readily bypass a leading-strand obstacle (Figure 5A). In support of this, PrimPol is required for the tolerance of impediments not efficiently bypassed by TLS, including chain-terminating nucleosides (Kobayashi et al., 2016), DNA secondary structures (Schiavone et al., 2016), R-loops (Šviković et al., 2019), cisplatin-induced adducts and hydroxyurea (Quinet et al., 2020), bulky DNA adducts that induce recombination (Piberger et al., 2020), and interstrand crosslinks (ICLs) (González-Acosta et al., 2020, Preprint). Indeed, although Rev1 is involved in bypass of G4-quadruplexes (G4s)—DNA secondary structures formed by Hoogsteen base-pairing between guanines—containing long loops (Schiavone et al., 2014), PrimPol is required for tolerance of G4s that are more thermodynamically stable (Schiavone et al., 2016). Moreover, on the fly TLS by Pol *η* was recently found to be the primary mechanism to recouple replisomes in response to UV damage in human fibroblasts, while PrimPol compensated for loss of Pol *η*, with the resulting ssDNA gaps filled in by template switching (Benureau et al., 2020, Preprint). Therefore, the relative efficiency of TLS bypass of a given impediment vs. repriming by PrimPol may dictate pathway choice. Although importantly, TLS can also function to fill in the ssDNA gap generated after repriming (Figure 5B) (Daigaku et al., 2010; Karras and Jentsch, 2010).

Following repriming, the nascent primer is likely extended by Pol *δ* to promote recoupling, as observed following TLS (Figure 5B) (Guilliam and Yeeles, 2020b). Interestingly, PolDIP2 (Pol *δ*-interacting protein 2, PDIP38) interacts with Pol *δ*, PCNA, and PrimPol, enhancing the polymerase activity of the latter (Maga et al., 2013; Guilliam et al., 2016; Kasho et al., 2021). PolDIP2 might therefore promote primer extension by PrimPol before also coordinating a switch to Pol *δ* for recoupling. A number of TLS polymerases also interact with PolDIP2 and loss of the protein causes a decrease in TLS *in vivo* (Tissier et al., 2010; Maga et al., 2013; Tsuda et al., 2019). However, whether PolDIP2 coordinates a switch back to Pol *δ* or another aspect of TLS or repriming in higher eukaryotes remains to be determined.

## Fork Reversal

**FIGURE 6:**
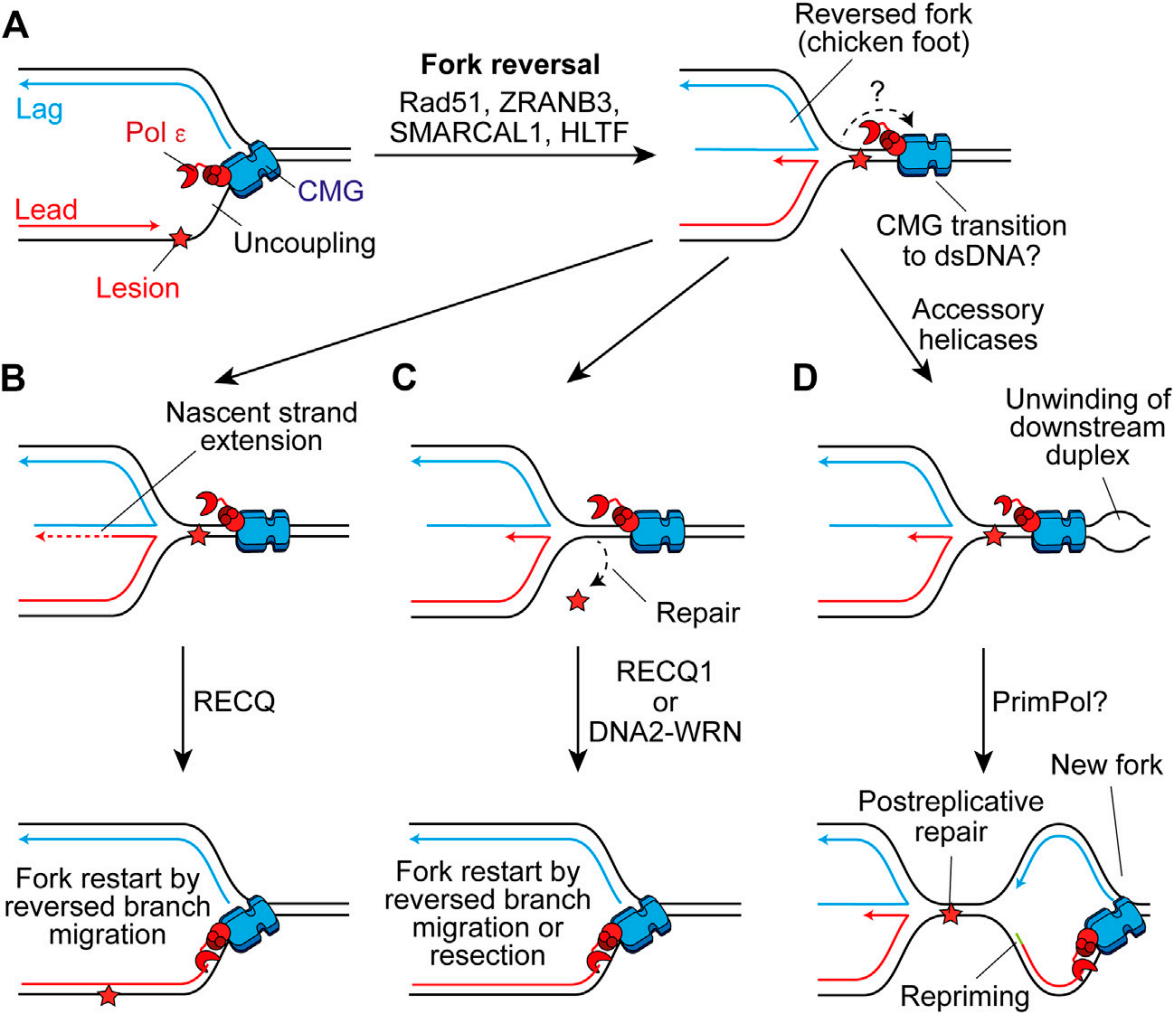
Fork reversal may maintain or restart replisome progression past leading-strand impediments. **(A)** Following uncoupling, fork reversal can be catalysed by Rad51, ZRANB3, SMARCAL1, and HLTF to generate a four-way chicken foot structure in a process which is not currently well understood. The fate of CMG here is not clear, one possibility is that it transitions on to duplex DNA downstream of the lesion. **(B)** The nascent lagging strand could serve as a template for extension of the nascent leading strand. Subsequent reversed branch migration, catalysed by RECQ1, may support fork restart if the extended 3′ end of the nascent leading strand is relocated downstream of the lesion. CMG may then transition back onto ssDNA to restart canonical replication. **(C)** Alternatively, fork reversal may place the lesion in a dsDNA context to permit repair, before resetting of the fork by RECQ1 or resection of nascent strands by DNA2-WRN. Again, CMG may transition back onto ssDNA to restart rapid fork progression. **(D)** Another possibility is that accessory helicases unwind the parental duplex downstream of the impediment following fork reversal. This may allow the establishment of a new replication fork which would require repriming of the leading strand. This would leave the chicken foot structure behind the new fork to be resolved following postreplicative repair of the lesion.

Template switching is a homologous recombination (HR)-like mechanism that promotes damage tolerance by using the undamaged nascent strand of the sister chromatid as a template (Branzei and Szakal, 2016). It can function postreplicatively to fill ssDNA gaps on the lagging strand, or following repriming on the leading strand by PrimPol (Piberger et al., 2020). However, template switching may also occur at the replication fork through fork reversal (Berti et al., 2020). Here, the fork regresses by annealing the nascent daughter strands, generating a four-way “chicken foot” structure (Figure 6A). This may allow extension of the nascent leading strand by using the undamaged nascent lagging strand as a template (Figure 6B), or place the damaged parental strand in a dsDNA context to permit canonical repair before fork restart (Figure 6C) (Berti and Vindigni, 2016).

Fork reversal requires Rad51 which binds ssDNA to promote strand invasion during HR (Scully et al., 2019) and partially replaces RPA on the ssDNA exposed by uncoupling, or nascent strand resection, to promote reversal (Berti et al., 2020). How these short stretches of Rad51 separated by RPA—termed metastable Rad51 filaments—are able to promote fork reversal is not currently clear (Berti et al., 2020). However, intriguingly this is not dependent on the enzymatic strand exchange activity of Rad51 (Mason et al., 2019). Additionally, following monoubiquitination by Rad6-Rad18, PCNA can be polyubiquitylated (Ripley et al., 2020). In yeast, Rad5 serves as the E3 ubiquitin-ligase (Hoege et al., 2002) and also has helicase activity that can directly reverse forks (Blastyák et al., 2007). Humans have two Rad5-related proteins, HLTF and SHPRH, both of which can polyubiquitylate monoubiquitinated PCNA, with HLTF shown to catalyse fork reversal *in vitro* (Unk et al., 2006; Blastyák et al., 2010; Kile et al., 2015). Two additional translocases also mediate fork reversal in humans, ZRANB3 and SMARCAL1, which are recruited by polyubiquitylated PCNA and RPA-ssDNA respectively (Ciccia et al., 2012; Bétous et al., 2013; Kolinjivadi et al., 2017; Taglialatela et al., 2017). These translocases fulfill non-redundant roles and might therefore contribute to different steps or be required in different contexts (Berti et al., 2020).

Although the generation of reversed forks does not require stable Rad51 filaments, BRCA2-mediated stable Rad51 filaments are required to protect reversed forks from nucleolytic degradation following their formation (Schlacher et al., 2011; Berti et al., 2020). Forks can subsequently be restarted by unwinding and controlled resection by WRN-DNA2 (Thangavel et al., 2015) or through reversed-branch migration by RECQ1 (Figures 6B,C) (Berti et al., 2013). Alternatively, a protected reversed fork could await resolution by the arrival of the convergent fork. However, prolonged fork reversal or de-protection can lead to processing by structure specific endonucleases, producing a broken fork (Berti et al., 2020). This can be rescued by break-induced replication, whereby strand invasion by the broken parental strand restarts replication but in an error prone and conservative manner that does not rely on a canonical replisome (Kramara et al., 2018).

Reversed forks have been directly detected using transmission electron microscopy (TEM) in human cells treated with a range of genotoxic agents, suggesting it is a universal response to replication stress (Zellweger et al., 2015). In support, fork reversal has been observed as an ATR-dependent global response to ICLs, even at forks not directly challenged by damage, to slow replication and promote repair (Mutreja et al., 2018). However, ATR phosphorylation of SMARCAL1 has also been shown to limit fork remodeling (Couch et al., 2013). Moreover, a recent study of human fibroblasts failed to detect any evidence of fork reversal by TEM in response to UV damage, even in the absence of Pol *η*, with repriming instead compensating for loss of TLS (Benureau et al., 2020, Preprint). In yeast, reversed forks were observed in repriming (Fumasoni et al., 2015) and checkpoint (Sogo et al., 2002; Lopes et al., 2006) mutants in response to bulky DNA lesions. A recent report showed that ATR-dependent up-regulation of PrimPol in cancer cells following multiple doses of cisplatin treatment suppresses fork reversal, revealing a competition between the two mechanisms (Quinet et al., 2020). This may be because PrimPol limits uncoupling and RPA-ssDNA is a prerequisite for fork reversal (Zellweger et al., 2015). Therefore, fork reversal and repriming likely compete when on the fly TLS cannot promote efficient bypass, with cell type, the nature of the replication impediment, and complex ATR-orchestrated responses potentially dictating which pathway is favored.

The precise details of fork reversal remain to be determined. In particular, it is not clear what happens to the replisome during this process. In bacteriophage T4, the replicative helicase must dissociate to permit remodeling of the fork (Manosas et al., 2012). However, in eukaryotes reloading of MCMs is inhibited in S phase to prevent re-replication (Chen and Bell, 2011; Frigola et al., 2013). To function as a mechanism to restart or maintain progression of a replisome, fork reversal must not cause dissociation of CMG. One possibility is that uncoupled CMG traverses onto dsDNA downstream of the stalled fork junction, before moving back onto ssDNA to restart replication when the canonical fork is restored (Figures 6A–C). Single-molecule analyses recently demonstrated that MCM can transiently open to transition from ssDNA to dsDNA, before diffusing back again to reform a functional replisome (Wasserman et al., 2019). This is not dependent on the Mcm2–5 gate used for loading during origin licensing, but does require Mcm10 which is essential for CMG activation. Here, Mcm10 may prevent dissociation of CMG upon opening of the ssDNA gate. However, Mcm10 also inhibits fork reversal by SMARCAL1 *in vitro* (Mayle et al., 2019). Moreover, in *Xenopus* egg extracts when CMG runs onto dsDNA at a lagging-strand nick it is ubiquitylated and removed by the same pathway used during termination (Vrtis et al., 2021). It is currently unclear how CMG encircling dsDNA to permit fork reversal would resist unloading by the same mechanism. Alternatively, CMG may rapidly reengage ssDNA downstream of the stalled fork junction, aided by accessory helicases (Figure 6D). Such a mechanism would require repriming and leave behind the chicken foot structure for resolution in a postreplicative manner (Berti et al., 2020). In either case, it is unclear what would trigger uncoupled CMG to transition from ssDNA to dsDNA. Indeed, in reconstitution experiments uncoupled CMG continues to unwind the parental template for multiple kb downstream of a CPD (Taylor and Yeeles, 2018, 2019).

## Traversal of Impediments to CMG Translocation

**FIGURE 7:**
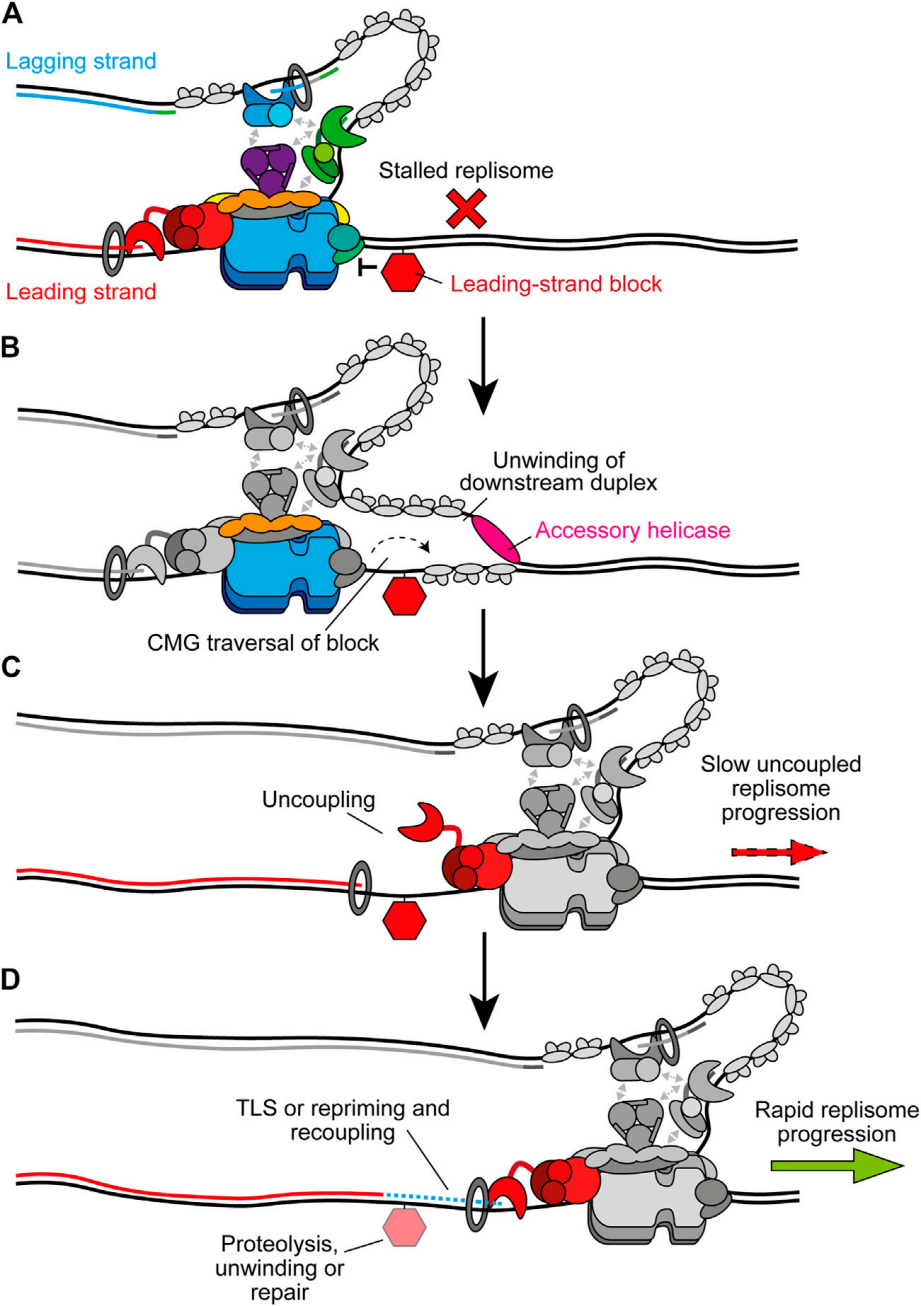
Bypass of CMG-blocking impediments on the leading strand. **(A)** Bulky leading-strand impediments including DPCs, ICLs, and preformed G4s can pose a block to CMG translocation, causing stalling of the replisome. **(B)** CMG traversal of the impediment is aided by transient opening of the MCM2-7 ring and accessory helicases which unwind the downstream parental duplex. The specific accessory helicase involved depends on the nature of the lesion, see text for details. Note that the accessory helicase may act on either the leading or lagging strand. Other replisome components are greyed out for clarity. **(C)** Following CMG traversal of the impediment, the replisome is uncoupled and fork progression is slow. **(D)** Recoupling first requires resolution of the impediment and/or extension of the leading strand past the damage site. Alternatively, leading-strand synthesis may be reinitiated downstream by repriming before postreplicative repair of the damage. In the case of DPCs, proteolysis occurs before TLS and recoupling. G4s are unwound by FANCJ to promote bypass and recoupling. ICL bypass requires PrimPol-mediated repriming before recoupling, leaving an X-shaped structure for postreplicative repair. Upon recoupling, rapid fork rates resume. Recoupling is indicated by a blue dotted line and Pol ε synthesis is shown in red.

Instead of causing uncoupling, bulkier replication impediments can directly block CMG translocation (Figure 7A). One example are DNA-protein crosslinks (DPCs), chromatin-bound proteins which are covalently crosslinked to DNA by chemotherapeutics or endogenous aldehydes (Stingele et al., 2017). Since dsDNA enters CMG a short distance, with strand separation occurring at the bottom of the ZF sub-ring (Yuan et al., 2020a; Baretić et al., 2020), DPCs might be expected to stall replisomes regardless of which template strand they are located in. However, in *Xenopus* extracts replisome progression is stalled much more significantly by a streptavidin block in the leading strand compared to the lagging strand (Fu et al., 2011). Purified yeast CMG is stalled by a streptavidin block located in either strand (Langston and O’Donnell, 2017), but can bypass the lagging-strand block in the presence of Mcm10 (Langston et al., 2017). Here, Mcm10 is hypothesised to bind the N tier of CMG to isomerise it to a steric exclusion model capable of bypassing impediments on the lagging strand (Langston et al., 2017). It is unknown if Mcm10 remains stably bound to the replisome following initiation or if it interacts dynamically when required.

Recent experiments in *Xenopus* extracts have revealed that the accessory helicase RTEL1 also stimulates bypass of a lagging strand DPC, although bypass still occurs in its absence (Sparks et al., 2019). Meanwhile, CMG stalling at a leading strand DPC is more extended and more sensitive to loss of RTEL1. Here, RTEL1 generates ssDNA downstream of stalled CMG to facilitate DPC bypass by transient opening of the Mcm2-7 ring, potentially aided by Mcm10 (Figure 7B) (Sparks et al., 2019; Wasserman et al., 2019). Single-molecule experiments confirmed that CMG bypass occurs before DPC degradation by the specialised protease SPTRN/DVC1 (Wss1 in yeast) (Sparks et al., 2019). Interestingly, upon collision the replisome facilitates ubiquitylation of the DPC by the E3 ubiquitin ligase TRAIP to promote proteolysis after CMG bypass. Ubiquitylation of the DPC also aids CMG bypass (Sparks et al., 2019). TRAIP is proposed to bind the leading edge of the replisome to fulfill this role which also positions it to ubiquitylate an adjacent CMG after fork convergence at an ICL (Wu et al., 2019, 2021). Following bypass, CMG progression is initially slow due to uncoupling (Figure 7C). TLS after DPC proteolysis subsequently recouples the fork (Figure 7D) (Duxin et al., 2014; Sparks et al., 2019).

ICLs also represent a block to replisome progression. In *Xenopus* extracts, ICL repair occurs following fork convergence at the lesion (Zhang et al., 2015). Here, TRAIP-dependent ubiquitylation of CMG stimulates recruitment of the NEIL3 glycosylase to resolve psoralen and abasic ICLs (Wu et al., 2021). If NEIL3 fails to unhook the crosslink, continued ubiquitylation of CMG by TRAIP triggers replisome disassembly to permit repair of the ICL by the Fanconi anemia pathway (Wu et al., 2021). Since both pathways depend on fork convergence, they cannot be considered as mechanisms to maintain progression of individual replisomes. However, studies in avian and mammalian cells suggest CMG can also traverse ICLs and maintain progression via an alternative mechanism (Huang et al., 2013, 2019; Ling et al., 2016; Mutreja et al., 2018). Here, FANCM associates with CMG in a FANCD2 and ATR-signalling dependent manner which triggers release of GINS from the replisome (Huang et al., 2019). FANCM translocase activity and loss of GINS may then promote opening of the Mcm2-7 ring and allow traversal of the ICL. FANCM also interacts with BTR (BLM/TOP3A/RMI1–2), and BLM helicase activity is important for ICL traverse (Ling et al., 2016). BLM might therefore play an analogous role to RTEL1 during DPC bypass by unwinding the parental strands downstream of the ICL (Ling et al., 2016). BTR also interacts with both RPA (Wu et al., 2018) and PrimPol (González-Acosta et al., 2020, preprint). Recent work demonstrated that PrimPol participates in ICL bypass, likely by reinitiating leading-strand synthesis to restore rapid fork rates (González-Acosta et al., 2020, preprint). Traversal would leave the X-shaped ICL structure behind the re-established replication fork to be repaired by the Fanconi anaemia pathway in a postreplicative manner (Lopez-Martinez et al., 2016).

Accessory helicases also assist the replisome in bypassing preformed G4 structures that can stall CMG when present in the leading-strand template (Lerner and Sale, 2019). Experiments in *Xenopus* extracts have recently delineated a mechanism for G4 bypass similar to that employed for DPC traversal (Sato et al., 2020, preprint). Following CMG collision with a G4, the accessory helicase DHX36 unwinds the parental duplex downstream of the structure. This allows CMG to bypass the G4 without unwinding through opening of the Mcm2-7 ring (Sparks et al., 2019; Wasserman et al., 2019). A second helicase, FANCJ, then assists G4 unwinding to permit recoupling of the leading strand to the replisome. There is partial redundancy between these two helicases for CMG bypass and notably the requirement for both is abolished by a convergent fork (Sato et al., 2020, preprint). DHX36 and FANCJ likely facilitate bypass in this context instead of RTEL1 due to their high affinity for G4s (Wu et al., 2008; Giri et al., 2011). G4s can also be bypassed by another recently identified mechanism (Lerner et al., 2020). Here, the replisome plays an active role in G4 resolution facilitated by a DNA binding domain in Timeless that has a strong preference for G4s. This allows Timeless to detect G4s in the vicinity of the replisome and promote their unwinding by DDX11, an accessory helicase that interacts with and is stimulated by Timeless (Lerner et al., 2020). The recent structure of Tof1, the yeast homologue of Timeless, in complex with CMG revealed that it binds ahead of the replisome, positioning it in an ideal location to detect G4s in the unwound parental duplex (Baretić et al., 2020). Importantly, G4s can adopt many different conformations and therefore these two mechanisms may favor different subsets of these.

## Summary

Aided by advances in biochemical systems, structural approaches, and single-molecule techniques, our understanding of the architecture of the replisome during unperturbed progression, the response of the replisome to impediments, and the DDT mechanisms employed to overcome these has increased greatly over recent years. In particular, these studies have revealed that DDT mechanisms function not only to relieve polymerase stalling, but also to maintain CMG translocation in the presence of obstacles previously thought to pose a complete block to template unwinding. Here, the combined action of transient opening of the MCM ring, unwinding of the parental duplex by accessory helicases, and recoupling of the nascent leading strand by classic DDT mechanisms has expanded the range of obstacles considered able to be tolerated by the replisome (Sparks et al., 2019; Wasserman et al., 2019; González-Acosta et al., 2020; Sato et al., 2020, preprint). Meanwhile, the chronology of pathway choice for recoupling leading strand replication is beginning to be deciphered. Recruitment of Pol *δ* to the leading strand appears to be the primary mechanism of recoupling (Guilliam and Yeeles, 2020b, 2021). When Pol *δ* is unable to fulfill this role, on the fly TLS is triggered by PCNA monoubiquitination and/or Rev1 recruitment to prevent prolonged uncoupling (Guilliam and Yeeles, 2020b). At bulkier impediments, or when TLS is inactivated, repriming and fork reversal compete to restore canonical replisome progression (Benureau et al., 2020, preprint; Quinet et al., 2020). In the case of repriming, the resulting ssDNA gaps can be filled in by postreplicative TLS or template switching, as will always occur on the lagging strand.

Many of these mechanisms offer potential targets for future chemotherapeutics. In particular, the role of TLS in both chemoresistance and mutagenesis has made TLS inhibitors a potentially promising future anti-cancer tool (Nayak et al., 2021). Further advances in biochemistry and structural biology will be key to fully understanding the mechanisms for maintaining replisome progression and how these can be exploited for disease treatment.
